# Whole exome sequencing for identifying rare genetic variants related to idiopathic granulomatous mastitis

**DOI:** 10.1007/s10067-025-07343-w

**Published:** 2025-02-24

**Authors:** Leyla Ozer, Hande Koksal

**Affiliations:** 1 Mikrogen Genetic Diagnosis Center, Reşit Galip Street 18/1 Çankaya, Ankara, Turkey; 2 Faculty of Medicine, Ardıçlı, Department of General Surgery Selcuk University, Celal Bayar Cd. No:313, Konya, Turkey

**Keywords:** Autoinflammation, Autoimmunity, Genetics, Granulomatous inflammation, Idiopathic granulomatous mastitis, Whole exome sequencing

## Abstract

**Backgrounds:**

To reveal rare genetic factors that cause susceptibility to idiopathic granulomatous mastitis (IGM).

**Methods:**

Whole exome sequencing (WES) was performed in 30 patients with histopathologically diagnosed idiopathic granulomatous mastitis. WES analysis mainly focused on 317 genes linked to autoimmunity, autoinflammation, and immune dysregulation.

**Results:**

A total of 141 variants were detected in 100 genes. The 40% (12/30) of patients had pathogenic or likely pathogenic variants. The pathogenic/likely pathogenic variants were 10.6% of all variants, and the rest of the variants (89.4%) were classified as VUS. Most of the variants were heterozygous (97.2%) only 4 variants (2.8%) were homozygous. Pathogenic/likely pathogenic variants were detected in FCGR1A, MPO, F5, IL36RN, CLUH, C9, NAXD, PROC, THRB, IFI30, COQ2, RNASEH2B, and SLC29A3 genes. The highest number of variants were detected in UNC13D, VPS13B, EPHB4, NLRP12, TCIRG1, TOM1, IRF9, and PIK3CG.

**Conclusion:**

Up to date, our study is the first whole exome sequencing study of IGM patients which aims to find out the rare variants related to etiopathogenesis of the disease. We identified 141 single nucleotide variants of 100 genes, and most of these variants were found in innate immunity-related genes. The current study provides clues for identifying the etiologic factors and designing further functional studies in this rare disease with unknown etiopathogenesis.

**Table:** 

**Key Points** •*Autoimmunity/autoinflammation-related genetic factors are blamed for etiopathogenesis of idiopathic granulomatous mastitis (IGM).* •*Mutation in genes related to innate immunity, especially in macrophage functions and phagocytosis, may lead to IGM susceptibility.* •*Potential candidate genes for genetic susceptibility to IGM may shed light for new treatment options*.

## Introduction

Idiopathic granulomatous mastitis (IGM) is a rare, benign, chronic mammary gland inflammation with unknown etiopathogenesis [1, 2]. Granulomatous mastitis and breast cancer may present similar clinic-radiologic findings [3]. Tuberculosis mastitis is the most common cause of granulomatous breast lesions among others [4]. Other common etiological factors responsible for the granulomatous lesions in the breast are autoimmune diseases such as Crohn’s disease, Wegener’s disease, and sarcoidosis [5, 6]. The diagnosis of IGM is almost a matter of exclusion. The clinical findings of IGM are inflammatory breast mass or masses, pain, inflammatory skin changes, lymphadenopathy, or nipple discharge. Recurrent episodes were reported in most of the cases [2]

The IGM is a granulomatous inflammation with non-caseating granuloma and micro abscess formation centered in the lobules [6]. Granulomas are defined as organized clusters of macrophages and other immune cells. Numerous stimuli (infectious agents and foreign bodies), as well as many inflammatory and autoimmune diseases of unknown etiology, can promote granuloma formation [7]. Some of the granulomas are formed in response to infectious agents to control infection by preventing the spread of these microorganisms [4]. Granulomas generally occur when agents that are foreign to the body, such as infectious agents and foreign bodies (like self-antigens), cannot be eradicated by macrophages [5, 7, 8]. Granuloma formation begins with the failure of phagocytosis upon the first stimulus [7]. During the progression of granulomatous inflammation, macrophages are transformed into epithelioid and giant multinucleated cells (Langhans cells). Loss of phagocytosis function will cause antigen expression on the surface of macrophages [5, 7]. These antigens activate Th-lymphocytes, and helper T cells (Th1) release chemokines and cytokines to recruit macrophages to the site of inflammation. The immune mechanisms of infectious granulomas are clearly understood however the information about the underlying factors in noninfectious granulomatous inflammation are limited [5, 9, 10]. Autoimmune factors are blamed for the etiology of noninfectious granulomatous inflammation. Self-antigens are sometimes labeled as foreign bodies and trigger the granuloma formation due to inadequate innate immunity response [8].

Many factors such as autoimmunity, infections, hormonal abnormalities, trauma, obesity, and smoking are blamed in the etiology of IGM [1, 6]. This benign granulomatous inflammation of the mammary gland may be related to autoimmunity limited to the breast because typical extramammary findings (laboratory and clinical) of systemic autoimmunity were not reported in all IGM cases [6, 11]. The high incidence of the disease in particular ethnic groups suggests that genetic factors might have a role in susceptibility to IGM. The underlying genetic causes of IGM have not yet been elucidated. Since autoimmune factors were thought to be the most likely etiological factor in noninfectious granulomatous inflammation, here it was aimed to investigate the possible genetic variants by focusing on the genes responsible for the immune response. The aim of this study is to reveal rare genetic factors that cause susceptibility to IGM.

## Methods

### Patient selection

In this cross-sectional study, the diagnosis of IGM was made by clinical, radiological, and pathological examination. Pathologically IGM was defined as a non-caseating granulomatous inflammatory reaction centered on lobules, granulomas composed of epithelioid histiocytes, Langhans giant cells accompanied by lymphocytes, plasma cells, and occasional eosinophils within and around lobules [2]. Also, Ehrlich-Ziehl–Neelsen staining is also applied to rule out tuberculosis at the diagnosis of IGM.

Between January 2023 and January 2024, the patients with histopathologically diagnosed granulomatous mastitis were included in this study after obtaining written informed consents. Inclusion criteria for this study were having a pathological diagnosis of IGM and agreeing to participate in the study. The only exclusion criterion was that the patient with IGM did not want to participate in the study.

The patients with any suspicious clinical or laboratory findings about other granulomatous lesion-forming diseases in the breast (tuberculosis, sarcoidosis, and Wegener’s granulomatosis) were excluded. Ethics Committee approval was received from Yüksek İhtisas University Faculty of Medicine, Clinical Research Ethics Committee (Approval Number:2021–06–25/1). The written informed consent to participate in the study was obtained from participants.

### Exome sequencing

Genomic DNA samples of 30 patients were isolated from peripheral blood using a DNA isolation kit (QIAamp DNA Blood Mini Kits, Qiagen, Hilden, Germany). Library preparation of sequencing was performed using the Twist Exome 2.0 kit. The generated library was sequenced on the MGI DNBSEQ-G400 NGS platform to achieve a minimum 20 × read depth for > 98% of the targeted bases. An average of 203,9055 variants were called per individual (range 169,077–238,734). Sequencing reads were then aligned against the human reference genome GRCh38 using the Franklin Genoox software (https://franklin.genoox.com/clinical-db/home). All detected variants were evaluated with respect to their pathogenicity and causality and were classified as pathogenic, likely pathogenic, and variant of unknown clinical significance (VUS) according to the American College of Medical Genetics and Genomics guidelines. WES analysis mainly focused on 317 genes linked to autoimmunity, autoinflammation, and immune dysregulation (Supplementary 1). The gene list was selected according to literature data [12–14].

All sequenced variants were filtered according to selection criteria for the purpose of identifying the variants that are most likely relevant to the pathogenesis of IGM. The detected variants were filtered according to their frequency in the general population and in our laboratory’s data. Only those variants with a frequency of ≤ 1% were selected. Variant frequencies were calculated via population frequency databases such as the Genome Aggregation Database (gnomAD /Exome Genome) and Exome Aggregation Consortium (ExAC). Several variant prediction tools including PolyPhen-2 and SIFT were used to estimate the functional effects of the variant.

### Pathway analysis

To evaluate protein–protein interactions of different genes Kyoto Encyclopedia of Genes and Genomes (KEGG) pathway analysis was used. The gene list was first submitted to the NIAID/NIH Database for Annotation, Visualization, and Integrated Discovery (DAVID) Bioinformatics Resources Analysis Wizard Tool (v2023q4). “OFFICIAL_GENE_SYMBOL” for the Identifier field, Homo sapiens for the Species field, and “Gene List” was selected under List Type. The submitted gene list was analyzed using the Functional Annotation Tool set, especially “KEGG Pathway” and the other pathway tools.

### Statistical analysis

In this study, descriptive statistical analysis was given. Frequency and percentage values were used for categorical variables. Median values with range were given for numerical variables.

## Results

Thirty patients were enrolled. The characteristics of the participants are presented in Table 1. The patients’ age range 23 to 54 years (median, 35 years). All patients were female. Ultrasonographic examination was evaluated as BIRADS 3 in all patients. Three of the patients had a history of erythema nodosum (EN) and 2 of them had arthritis at the same time. One of the patients with both EN and arthritis was refractory to steroids, had been treated with methotrexate, and is now in follow-up with azathioprine as maintenance therapy. One patient had arthritis only and another one had psoriasis. Newly diagnosed breast cancer was observed in one of the patients with EN. Two of the cases had bilateral IGM.

**Table 1:** The patients’ characteristics

**Characteristics**	*N* (%)
Unilateral location	28 (93.3)
Bilateral location	2 (6.6)
Breast abscess/mass	30 (100)
Axillary lymphadenopathy	3 (10)
Erythema nodosum	3 (10)
Arthritis	10 (3)
Breast cancer	1 (3.3)
Other disease (psoriasis, hyperthyroidism)	2 (6)
Radiological evaluation/ BIRADS 3	30 (100)
Response to steroid treatment	29 (96.7)
Negative microbiological culture	30 (100)

*BIRADS*, Breast Imaging Reporting and Data System

The patients are found to be carriers of 2–8 variants in genes related to autoimmunity, autoinflammation, and immune regulation (Table 2). The coverage achieved from whole exome sequencing is 95–99% at > 20 × . A total of 141 variants were detected in 100 genes. The 40% (12/30) of patients had pathogenic or likely pathogenic variants. The pathogenic/likely pathogenic variants were 10.6% of all variants and the rest of the variants (89.4%) were classified as VUS. Most of the variants were heterozygous (97.2%) only 4 variants (2.8%) had homozygous variants. Homozygous variants were detected in IFI30, LYST, DCLRE1C, and C4B genes. Nearly all variants were unique to each individual, except for the variants of MPO (c.2031-2A > C), which were found in two different individuals. Seventy-eight percent of variants were missense mutations, 17.1% of variants were splice mutations, 1.4% of variants were frameshift mutations, 1.4% of variants were stop gain mutations, 0.7% of variants were stop loss, and 1.4% were noncoding-regions. 40.2% of variants had an allele frequency of less than 0.01% in the gnomAD database, 41.6% of variants had an allele frequency of 0.01–0.5% in the gnomAD database 18% of variants were not reported in the gnomAD database. The highest number of variants were detected in UNC13D (6 variants), VPS13B (4 variants) and EPHB4, NLRP12, TCIRG1, TOM1, IRF9, and PIK3CG (3 variants). Pathogenic/likely pathogenic variants were detected in FCGR1A, MPO, F5, IL36RN, CLUH, C9, NAXD, PROC, THRB, IFI30, COQ2, RNASEH2B, and SLC29A3 genes.

**Table 2:** Rare pathogenic/likely pathogenic variants detected in patients with idiopathic granulomatous mastitis

Cases	Gene	Nucleotide change	Amino acid change	MAF (%)	Zygosity	Gene function	OMIM disease/inheritance
1	FCGR1A	c.274C > T	p.Arg92*	≤ 1	Heterozygous	Innate and adaptive immune response, phagocytosis, antigen presentation, clearance of immune complexes	IgG receptor I, phagocytic, familial deficiency of /NA
	MPO	c.2031-2A > C		≤ 1	Heterozygous	Innate immune response, phagocytosis	Myeloperoxidase deficiency/AR
	F5	c.5534A > G	p.His1845Arg	0.01	Heterozygous	Coagulation and immune response	Thrombophilia, susceptibility to/AD
2	MPO	c.2031-2A > C		≤ 1	Heterozygous	Innate immune response, phagocytosis	Myeloperoxidase deficiency/AR
3	IL36RN	c.338C > T	p.Ser113Leu	≤ 1	Heterozygous	Regulation of inflammation	Pustular psoriasis 14/AR
	CLUH	c.505C > T	p.His169Tyr	NA	Heterozygous	Regulation of inflammation, mitochondrial function	NA
4	C9	c.355 T > G	p.Cys119Gly	0.03	Heterozygous	Innate immune response, complement system	C9 deficiency/NA
5	COQ2	c.287G > A	p.Ser96Asn	< 0.01	Heterozygous	Regulation of inflammation, controlling the reactive oxygen species (ROS) production	Coenzyme Q10 deficiency/AR Multiple system atrophy, susceptibility to/ AD/AR
6	NAXD	c.176dupG	p.Val60fs	NA	Heterozygous	Regulation of inflammation, controlling the reactive oxygen species (ROS) production	Encephalopathy, progressive, early-onset, with brain edema and/or leukoencephalopathy/AR
	PROC	c.1106C > T	p.Pro369Leu	< 0.01	Heterozygous	Anti-inflammatory activity, anticoagulant activity	Thrombophilia 3 due to protein C deficiency/AD/AR
7	THRB	c.1357C > G	p.Pro453Ala	< 0.01	Heterozygous	Thyroid hormone receptor beta protein synthesis	Thyroid hormone resistance/AD/AR
8	IFI30	c.304G > A	p.Gly102Arg	0.01	**Homozygous**	MHC class II-restricted antigen processing, regulation of CD4 + T cell-mediated autoimmunity	NA
9	NAXD	c.1141C > T	p.Arg381Ter	< 0.01	Heterozygous	Regulation of inflammation, controlling the reactive oxygen species (ROS) production	Encephalopathy, progressive, early-onset, with brain edema and/or leukoencephalopathy/AR
10	COQ2	c.105C > A	p.Cys35Ter	NA	Heterozygous	Regulation of inflammation, controlling the reactive oxygen species (ROS) production	Coenzyme Q10 deficiency/AR Multiple system atrophy, susceptibility to/ AD/AR
11	RNASEH2B	c.529G > A	p.Ala177Thr	0.14	Heterozygous	Innate immune response	Aicardi-Goutieres syndrome 2/AR
12	SLC29A3	c.15dupA	p.Glu6fs	NA	Heterozygous	NA	Histiocytosis-lymphadenopathy plus syndrome/AR

*OMIM* online mendelian inheritance in man, *MAF* minor allele frequency, *NA* not available, *AR* autosomal recessive, *AD* autosomal dominant, *FCGR1A* Fc fragment of IgG receptor Ia, *MPO* myeloperoxidase, *F5* coagulation factor V, *IL36RN* interleukin 36 receptor antagonist, *CLUH* clustered mitochondria, *D*. discoideum, homolog of, *COQ2* coenzyme Q2, *COQ2* polyprenyltransferase, *NAXD* NAD(P)HX dehydratase, *PROC* proteın C, *THRB* thyroid hormone receptor, beta, *IFI30* interferon-gamma-inducible protein 30, *RNASEH2B* ribonuclease H2, subunit B, *SLC29A3* solute carrier family 29 (nucleoside transporter), member 3

## Discussion

In the current study, it was aimed to find out rare genetic variants relevant to immune processes which may be responsible for the IGM etiopathogenesis. Among 30 IGM cases, we detected rare pathogenic/likely pathogenic variants in FCGR1A, MPO, F5, IL36RN, CLUH, C9, NAXD, PROC, THRB, IFI30, COQ2, RNASEH2B, and SLC29A3 genes (Table 2). These genes were previously reported in immune processes such as autoimmunity and autoinflammation. Below, the possible effects of these 13 gene variants on the etiopathogenesis of IGM are discussed. Of the 13 genes with rare pathogenic/likely pathogenic variants associated with IGM in our study; 5 genes encode proteins which have roles involved in macrophage functions and phagocytosis (FCGR1A, MPO, F5, PROC, IFI30), 3 of 13 genes (NAXD, COQ2, CLUH) are related with mitochondrial function, 3 of the 13 genes (IL36RN, RNASEH2B, SLC29A3) are related with autoimmune processes and C9 gene is related with complement system.

In this study most of the detected genetic variants are localized in genes related to innate immunity, especially in macrophage functions and phagocytosis. Macrophages play an important role in the innate system and clearance of foreign substances via phagocytosis [5, 15]. The impaired phagocytosis function of macrophages is the first step of the formation of granulomatous inflammation [9, 10, 15]. The clearance of apoptotic cells decreased due to the weakening of the phagocytic function of macrophages and increased apoptotic cells leads to trigger the production of autoimmune antigens/antibodies and cause inflammation [9, 10, 15]. Five cases had pathogenic variants in FCGR1A, MPO, FV, PROC, and IFI30 genes which are responsible for macrophage functions and phagocytosis. FCGR1A is a member of the FCGR1 (CD64) gene family which has critical roles in immune functions such as phagocytosis, antigen presentation, degranulation, clearance of immune complexes, and cytokine production [16]. FCGR1A gene variants were associated with the development and severity of sarcoidosis where granulomatous inflammation takes place in the pathogenesis of the disease [16]. A previous RNA sequencing study revealed the gene expression profiles associated with granulomatous mastitis and they found that FCGR1A was the hub gene in immune system-related mRNAs and linked to 9 other genes associated with protein and IgG binding [17]. Therefore, FCGR1A may play a major role in the immune pathways associated with granulomatous mastitis. The presence of a heterozygous FV gene variant might be the potentiator of inflammation in IGM cases. The coagulation pathway is strongly linked to innate immunity and inflammatory processes. FV and PROC gene variants increase the thrombosis and complicate the inflammatory response [18, 19]. In our study, two cases have the same pathogenic variant in the MPO gene (c.2031-2A > C). MPO regulates neutrophil trafficking, phagocytosis, formation of extracellular traps, and MPO-triggered autoimmunity [20]. MPO gene mutations cause partial or complete myeloperoxidase deficiency which activates proinflammatory cytokines (IL-36), reduces the formation of neutrophil extracellular traps, and decreases the phagocytic function of neutrophils [21, 22]. MPO variants are genetic risk factors for autoinflammatory conditions [22]. Heterozygous and homozygous MPO variants were reported in several cases of generalized pustular psoriasis (GPP) and some of the cases had also one or two IL36RN gene variants and the other had only one or two MPO variants [23]. Our one case had both heterozygous MPO and IL36RN variants. According to these previous reports, MPO deficiency should likely be related to autoinflammatory and autoimmune responses via activating proinflammatory cytokines and impairing phagocytic functions of neutrophils. A homozygous IFI30 gene variant (c.304G > A, p. Gly102Arg) was identified in one patient. IFI30 gene encodes GILT enzyme which is expressed in antigen-presenting cells and plays a role in MHC class II-restricted antigen processing and regulates CD4 + T cell-mediated autoimmunity. Loss of function of the GILT enzyme causes high levels of superoxide levels, increased oxidative stress, and decreased mitochondrial function [24, 25]. Kosciuczuk et al. [26] showed the IFI30 expression changes by transcriptome profiling of Staphylococci-infected mammary gland parenchyma in cows. Based on this study’s results, te IFI30 gene plays a role in mammary gland immune defense via antigen processing and presentation. The detected homozygous IFI30 variant might also be evaluated as a potential risk factor for IGM etiopathogenesis.

The genetic variants related to mitochondrial functions were found in NAXD, CLUH, and COQ2 genes. Both NAXD and COQ2 have critical roles in mitochondrial metabolic pathways and controlling the reactive oxygen species (ROS) production. The over-production of ROS stimulates the pro-inflammatory cytokines and inflammatory processes [27, 28]. NAXD gene encodes NAD(P)HX dehydratase that has functions in mitochondrial metabolism and in metabolite repair system for removing the toxic NAD(P)HX side products [27]. COQ2 encodes the enzyme that is essential for COQ10 biosynthesis. COQ10 has an antioxidant function and several roles in electron transport, oxidative phosphorylation, and immune regulation via its anti-inflammatory role [29, 30]. CLUH is a negative regulator of proinflammatory cytokine secretion and loss of function of CLUH triggers the secretion of proinflammatory cytokines IL-6 and TNF-α and promotes inflammation. Reduced CLUH gene expression increases ROS production, reduces mitophagy and lysosomal function in macrophages, and was reported in ulcerative colitis [31]. Loss of functions of the CLUH gene might cause susceptibility to autoinflammation by inducing proinflammatory cytokines.

This current study shows the genetic changes in genes related to autoimmunity such as pathogenic variants of IL36RN, RNASEH2B, and SLC29A3 genes. It was shown that IL-36 expression is dysregulated in various autoimmune diseases such as psoriasis, rheumatoid arthritis, inflammatory bowel disease, systemic lupus erythematosus (SLE), and Sjogren’s disease [32]. IL36RN gene variants cause increased IL-36 levels and IL-36 activates the proinflammatory cytokines and inflammation [33–35]. Heterozygous and homozygous IL36RN c.338C > T (p. Ser113Leu) variants which were found in our study were reported in several GPP cases [36]. Most of the studies focused on treatment and pathogenesis of psoriasis revealed the presence of homozygous or heterozygous IL36RN variants in cases [34–36]. RNASEH2B gene is a member of RNASEH2 genes which prevents DNA from the damage. Heterozygous mutations in the RNASEH2 genes are also associated with SLE and systemic autoimmunity [37]. In our study, a pathogenic SLC29A3 gene variant was also detected. Recent studies have stated that SLC29A3 mutations are related to autoinflammation and autoimmunity due to the activation of inflammatory processes by mitochondrial and lysosomal dysfunction. SLC29A3 homozygous variant was reported in cases with systemic juvenile idiopathic arthritis and hyperferritinemia [37, 38].

In the current study, genetic variants associated with the complement system were also reported. Changes in the complement system have been reported in several autoimmune disorders such as rheumatoid arthritis, SLE, and inflammatory myositis. C9 deficiency has been reported in limited patients with dermatomyositis and susceptibility to various infections [39].

Polyarthritis and erythema nodosum (EN) are reported as extramammary findings of IGM. The frequency of co-existence with EN and IGM cases were reported as 4.4–6.6% in previous studies [40]. The presence of EN and arthritis commonly reported in autoimmune disorders, the response to immunosuppressive treatment, and the absence of infectious etiology in pathological evaluation give rise to the thought that autoimmunity and immune dysregulation may be related to the etiopathogenesis of IGM. In the present study, 4 of 30 cases had extramammary findings such as EN and arthritis. In cases with EN and arthritis, pathogenic variants of CLUH and IL36RN genes were detected. Two of the cases of EN had heterozygous UNC13D variants (VUS). One of them had also steroid resistant severe IGM and heterozygous PLCG2, SYK, IL2RA, CARD14, and FERMT3 gene VUS variants in addition to the UNC13D variant were detected. The highest number of variants were detected in the UNC13D gene. UNC13D regulates the number of T cells by destroying unneeded ones thus preventing cytokine production and inflammation. The loss of function variants (homozygous or heterozygous) of UNC13D is associated with susceptibility to autoimmune lymphoproliferative syndrome (ALPS) [41]. UNC13D variations were reported in autoimmune disorders like systemic juvenile idiopathic arthritis [42].

Case with psoriasis has homozygous DCLRE1C, heterozygous ZAP70, RFX5, AKT1, and NOD2 gene variants, and all of them were VUS variants. The NOD2 mutations can cause various conditions such as immune deficiencies, autoimmune and autoinflammatory conditions, and psoriasis [43]. Bercot et al. reported a case with granulomatous mastitis due to Corynebacterium kroppenstedtii which had a single nucleotide polymorphism within the NOD2 gene [44]. They postulated that impaired NOD2 function decreases phagocytic functions related to hypogammaglobulinemia and causes the formation of granulomatous mastitis [44]. Homozygous DCLRE1C gene variants cause severe combined immunodeficiency but rare homozygous variants were reported in cases with mild phenotype [45].

A case with hyperthyroidism had a heterozygous pathogenic variant in THRB gene which encodes thyroid hormone receptor protein. Heterozygous THRB gene mutations cause hyperthyroidism due to thyroid hormone resistance (OMIM ID:188750).

To our knowledge, the current study is the first study of whole exome sequencing in IGM patients to find out the rare variants related to IGM. Our study has some limitations like small number of cases, the structure of the control group, and the absence of segregation analysis. To give statistically significant results, further studies with a larger number of patients will be needed. Using public sequencing databases brings some difficulties like the contamination of the control group because the control datasets might include individuals with autoinflammatory or autoimmune conditions.

Some of the genes that were detected in the current study have been previously reported in several autoimmune/autoinflammatory diseases. Most of the detected genetic variants are localized in genes related to innate immunity, especially in macrophage functions and phagocytosis. The features of IGM (the absence of self-reactive antibodies and T-cells, immune response to own tissues) and the results of the current study state that IGM can be evaluated as an autoinflammatory disease. As each case carries 2–8 gene variants, the detected variants might have an additive effect on each other.

Currently, corticosteroids and, rarely, other immunosuppressive/immunomodulatory drugs are used in IGM treatment in patients resistant to corticosteroids [2]. The presence of genetic variants detected in our results supports autoimmunity and immune dysregulation in the etiopathogenesis of the disease. It may contribute to direct the treatment by means of making a decision in choice of alternative immunosuppressive and immunomodulatory drugs, especially in patients resistant to first-line treatment.

## Conclusion

In summary, this study revealed the potential candidate genes for predisposition to IGM. These 11 genes—FCGR1A, MPO, IL36RN, CLUH, C9, NAXD, IFI30, COQ2, RNASEH2B, SLC29A3, UNC13D—show significant promise as potential candidate genes for genetic susceptibility to IGM. Our findings will provide information about the molecular mechanisms underlying IGM pathogenesis and will shed light on future studies on the development of new treatment strategies. There are a limited number of genetic studies in this field, and new studies are needed to understand the genetic causes and molecular basis of the disease. Further researches with functional studies to understand gene expression will increase the benefits of the current study’s results.

## Data Availability

Data are available from the corresponding author upon reasonable request.
